# Microbiome mediation of infections in the cancer setting

**DOI:** 10.1186/s13073-016-0306-z

**Published:** 2016-04-18

**Authors:** Ying Taur, Eric G. Pamer

**Affiliations:** Infectious Disease Service, Department of Medicine, Memorial Sloan‐Kettering Cancer Center, 1275 York Avenue, New York, NY 10065 USA

## Abstract

Infections encountered in the cancer setting may arise from intensive cancer treatments or may result from the cancer itself, leading to risk of infections through immune compromise, disruption of anatomic barriers, and exposure to nosocomial (hospital-acquired) pathogens. Consequently, cancer-related infections are unique and epidemiologically distinct from those in other patient populations and may be particularly challenging for clinicians to treat. There is increasing evidence that the microbiome is a crucial factor in the cancer patient’s risk for infectious complications. Frequently encountered pathogens with observed ties to the microbiome include vancomycin-resistant *Enterococcus*, Enterobacteriaceae, and *Clostridium difficile*; these organisms can exist in the human body without disease under normal circumstances, but all can arise as infections when the microbiome is disrupted. In the cancer patient, such disruptions may result from interventions such as chemotherapy, broad-spectrum antibiotics, or anatomic alteration through surgery. In this review, we discuss evidence of the significant role of the microbiome in cancer-related infections; how a better understanding of the role of the microbiome can facilitate our understanding of these complications; and how this knowledge might be exploited to improve outcomes in cancer patients and reduce risk of infection.

## Cancer-related infections

Many patients with neoplastic disease are at increased risk for a variety of infections, either because of adverse effects from cancer treatment or because of the underlying cancer itself. The nature of these infections is frequently related to host insults such as immune suppression, anatomic defects, and epithelial barrier damage. Intensive treatments such as chemotherapy, radiation, and major surgery may each give rise to specific infectious risks. In response, broad-spectrum antimicrobials are commonly administered, which in turn have further shaped and altered the epidemiologic profile of cancer-related infections. As a result, management of infectious complications in patients with cancer is a unique and dynamic challenge for clinicians.

It is increasingly being recognized that the microbiome may be particularly relevant in many cancer-related infections. For example, infections in cancer patients more frequently involve or originate from the intestinal tract than those of non-cancer patients. Typical pathogens seen in cancer patients consist largely of microorganisms originating from the intestinal tract, such as *Escherichia coli*, *Klebsiella* spp., *Enterococcus*, viridans streptococci, and *Candida albicans* [1, 2]. This contrasts sharply with general hospitals, where *Staphylococcus aureus* is more typically the most common pathogen encountered, which preferably colonizes skin [3].

In this review, we examine the role of the microbiome in cancer-related infections. Many non-infectious ties have been made between cancer and the microbiome but will not be discussed here specifically, though some concepts may be overlapping. These include carcinogenesis [4–7], metabolism of immunosuppressants [8], and graft-versus-host disease in hematopoietic stem cell transplantation (HSCT) [9–11]. Here we focus on the microbiome’s relevance to cancer patients in terms of infectious complications and how the microbiome might be exploited to improve outcomes for these patients.

## Significance of the gut microbiome in cancer and infectious implications of a disrupted microbiome

In the intestinal tract, significant disruption of microbial populations due to cancer treatment may explain why the microbiome may be central to understanding the development of infectious complications. One patient group in which the microbiome has been well studied is patients undergoing allogeneic HSCT (allo-HSCT), a cancer treatment that simultaneously exposes patients to cytotoxic chemotherapy, total body irradiation, immunosuppressants, and broad-spectrum antibiotics. Examination of the intestinal microbiome of such patients through serially collected stool specimens at one cancer center demonstrated significant changes in the microbial population, marked by an overall reduction of microbial diversity [12]. Subsequent study of these patients showed that recipients with decreased gut microbial diversity soon after stem cell transplantation were, on average, more likely to die over the next 3 years than those with high gut microbial diversity, independent of other known mortality predictors in allo-HSCT, such as disease status, pre-transplant comorbidity, organ dysfunction, myeloablative intensity of treatment, and even antibiotic administration [13]. More specifically, low gut microbial diversity was primarily associated with transplant-related deaths (death not related to relapse or recurrence of the malignancy), suggesting that the gut microbiome’s association with overall mortality is largely related to complications of transplantation, namely opportunistic infections and graft-versus-host disease, where lymphocytes derived from transplanted stem cells attack host recipient tissues.

**Fig. 1:**
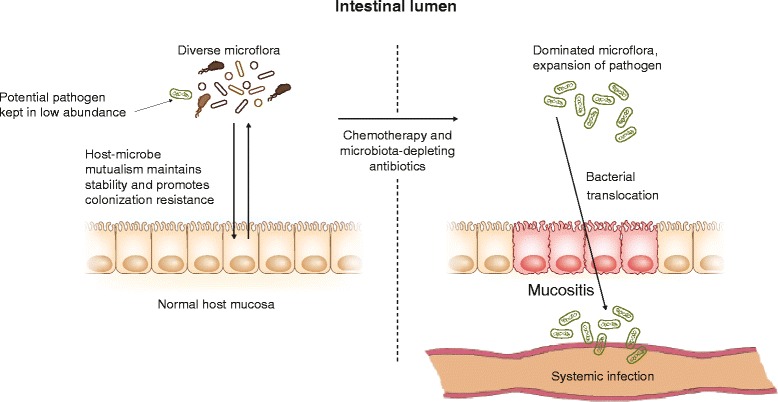
Disruption of the intestinal microbiota during cancer chemotherapy. Under normal circumstances (*left*), the healthy and diverse bacterial microflora and the host tissues promote stability and colonization resistance, preventing expansion of potential pathogens. Systemic chemotherapy (*right*) leads to mucosal barrier injury (mucositis). During this time, the microbiota is also disrupted, possibly by chemotherapy or by antibiotics that are given simultaneously, or because of decreased host control over microbial populations, or expansion of a pathogenic species due to mucosal inflammation. The microbiota is dominated by a single pathobiont, which can escape into the systemic circulation by translocation through damaged epithelial tissues. Spread beyond mesenteric lymph nodes particularly occurs as a result of failure of systemic immune defenses

Significant disruption of gut inhabitants may explain the observed importance of the microbiome in allo-HSCT. Under normal circumstances, a healthy intestinal microbiome is maintained and prevents infection by promoting colonization resistance, thus blocking overgrowth and expansion of rogue pathobionts, which typically exist as minority members in the microbiota (Fig. 1). This concept is not necessarily a new one and in fact was realized to have important implications for cancer treatment over four decades ago. The term colonization resistance was first used in 1971 by van der Waaij [14], who observed that intestinal flora containing anaerobic bacteria can resist colonization by *E. coli*, *Klebsiella pneumoniae*, and *Pseudomonas aeruginosa*.

At the time, patients with leukemia and other malignancies were being treated with increasingly effective but intensive chemotherapeutic regimens. Patients were highly susceptible to infectious complications and prevention of these infections became an important focus. This led to the use of strict protective isolation of patients in sterile systems and routine decontamination of the gastrointestinal tract and skin. These programs attempted to keep patients under strict gnotobiotic conditions: sterile isolation rooms with laminar air flow units were used, all food and water were sterilized, and skin and gut decontamination was routinely performed using topical and non-absorbable antibiotics [15]. Although there seemed to be some initial evidence of benefit, subsequent larger studies examining these measures failed to demonstrate sufficient benefit to warrant continuation of these massive efforts [16, 17], and these measures fell out of favor at most cancer institutions.

The concept of colonization resistance gave rise to the notion that infections related to cancer treatment could be better prevented by a more judicious, selective inhibition of microbes, rather than total decontamination. Attempts at prevention of infection turned to selective decontamination of the digestive tract, in which more targeted antibiotics were administered that could selectively remove potential aerobic pathogens yet retain colonization resistance against new pathogens. This approach made use of antibiotics that have little impact on anaerobic bacteria, such as nalidixic acid, trimethoprim-sulfamethoxazole, or polymyxin B [15]. Later, fluoroquinolones such as ciprofloxacin and levofloxacin were also widely used for selective prevention of infection during cancer treatment [18]. These prophylactic approaches provided more effective protection and continue to be practiced today.

More recent work suggests that promotion of colonization resistance occurs through a variety of mechanisms. These include direct inhibition of pathogens by beneficial microbes, through the production of bacteriocins, and indirect mechanisms involving the host, such as activation of immune defenses (for example, nucleotide-binding oligomerization domain-containing protein 2 (NOD2), which is involved in the immune response to bacterial infection) or enhancement of epithelium-derived antimicrobial peptides (for example, regenerating islet-derived III gamma (RegIIIγ)) [19]. In cancer, damage is incurred to commensal bacteria, the immune system, and gut epithelium, which explains the observed loss of colonization resistance and subsequent enhanced susceptibility to infection in afflicted patients.

## Chemotherapy and bloodstream infections due to mucosal barrier injury

Cytotoxic chemotherapy remains one of the mainstays of treatment for a variety of cancers and may be given either alone or as part of HSCT. As an adverse effect, it causes varying degrees of damage to hematopoietic cells, which commonly leads to neutropenia, which places the patient at risk for certain infections. Although various sources are possible, concurrent damage to the intestinal mucosa is the singular most common source of infection in neutropenic patients. Mucosal barrier injury by chemotherapy is the earliest and most frequently encountered breach in host defenses against pathogenic microorganisms.

Sonis [20] described the dynamics of mucosal barrier injury (also known as mucositis) as a sequential series of stages, involving free radical generation, induction of inflammation and apoptosis, signal amplification leading to more inflammation and apoptosis, discontinuity of the epithelial barrier leading to translocation of microorganisms, and subsequent spontaneous healing through cell proliferation. Translocation of intestinal microorganisms to the systemic circulation manifests as bloodstream infection, which can be life-threatening if sepsis ensues. Mucosal barrier injury and exposure to antimicrobial agents probably explains the emergence of most infections arising in neutropenic patients.

Despite the extensive damage to the gastrointestinal tract, symptoms are frequently not localized; fever may often be the only symptom manifested. In current clinical practice, fever in the setting of neutropenia is sufficient to warrant prompt initiation of empiric systemic antibiotics. Antibiotics are primarily selected to target potentially pathogenic bacteria and fungi that may reside in the gut. These include aerobic Gram-negative bacteria such as *E. coli*, *K. pneumoniae*, or *P. aeruginosa*, Gram-positive bacteria such as viridans streptococci and *Enterococcus* spp., and fungi such as *Candida albicans*. Although these oxygen-tolerant pathobionts are thought to originate from the intestinal tract, they exist in low relative abundance within the gut lumen under normal circumstances. Notably, obligate anaerobic bacteria, which are typically far more abundant in the large intestine and other parts of the intestinal tract, are rarely seen as bloodstream infections in this setting. Antibiotics with anti-anaerobic activity are therefore not required in the empiric treatment of fever and neutropenia, which is reflected in current clinical practice standards [21].

Systemic bloodstream infection due to mucosal barrier injury and subsequent bacterial translocation has been shown more recently to be closely related to dynamic changes in the intestinal microbiome. In one study of 94 patients undergoing allo-HSCT at a transplant center, serial fecal specimens showing loss of microbial diversity demonstrated a concurrent increased abundance and overgrowth of certain pathogenic bacteria [12]. The most common bacteria observed were vancomycin-resistant *Enterococcus* (VRE), Enterobacteriaceae such as *E. coli* and *Klebsiella* spp., and viridans streptococci. Interestingly, these organisms were the most common bloodstream isolates recovered from patients undergoing allo-HSCT at this institution [22–24]. Expansion and domination of these pathogens in the gut was associated with subsequent systemic infection with the corresponding pathogen in blood; patients who developed VRE bloodstream infection had a preceding domination of the intestinal microbiome by VRE and patients who developed Gram-negative bloodstream infections had a preceding domination by proteobacteria (the phylum of bacteria containing many known aerobic Gram-negative pathogens).

This provided confirmation that bloodstream infections during neutropenia arise largely from a gut source and that translocation of bacteria is preceded by a transformative process in the gut microbiome, in which colonization resistance is promptly lost, leading to overgrowth by a single species (Fig. 1). This provides a potential explanation for why anaerobes are not commonly encountered in systemic infections, despite their overwhelming presence in the gut under normal circumstances. If bloodstream infections during fever and neutropenia occurred merely because of a cancer-treatment-related breach in the intestinal mucosa, one might have expected a greater predominance of anaerobic infections.

These microbial changes took place a median of 7 days before the onset of detectable bacteremia, raising the question of whether examination of the fecal microbiota could forewarn of impending systemic infection in these patients. Perhaps not surprisingly, administration of antibiotics, specifically those with anti-anaerobic activity, was correlated with subsequent expansion of pathogenic bacteria [12]. Other factors, such as chemotherapy, may contribute to disruption of the microbiota, either by damaging host mechanisms that would normally help to maintain microbial populations and enhance colonization resistance or through direct killing of bacteria. Although currently not known, it may be the case that preservation or repair of a functionally intact microbiota may help to prevent the progression of mucosal barrier injury. Van Vliet and colleagues [25] proposed several mechanisms by which intestinal bacteria might serve to interfere with damage to intestinal tissues, building on the original Sonis [20] model of mucositis. These proposed mechanisms include: (1) modulation of inflammation and oxidative stress through a variety of mechanisms by beneficial members such as *Bacteroides thetaiotaomicron*, *Clostridium* cluster XIVa, and *Faecalibacterium prausnitzii*; (2) attenuation of intestinal permeability by members such as bifidobacteria and lactobacilli, which increase tight junction expression; (3) maintenance of the mucus layer, for example, by various *Lactobacillus* species, which upregulate mucin production; (4) stimulation of epithelial repair through butyrate and other factors generated by symbiotic bacteria; and (5) regulation of immune effector molecules such as RegIIIγ and IgA, which promote intestinal homeostasis and colonization resistance.

## *Clostridium difficile* infection

*C. difficile* infection has perhaps one of the clearest ties to the microbiome, as it is known to result from disruption of normal intestinal bacteria following antibiotic administration and other perturbations of the gut flora. In certain cancer patient populations, rates of *C. difficile* infection are particularly high. This may be related to a combination of factors, including frequent use of broad-spectrum antibiotics, immune suppression, prolonged or frequent hospitalizations, and chemotherapy, which has been observed to cause *C. difficile* infection by itself [26, 27].

In patients undergoing treatment with HSCT, high rates of *C. difficile* infection have been observed, typically ranging from 12 to 30 % [28–32]. These rates far exceed those in the general patient population, where incidence is generally less than 1 % [33]. This may be a reflection of the extreme degree of microbial dysbiosis experienced by these patients over the course of transplantation.

In one study of *C. difficile* infection in patients hospitalized to undergo HSCT, examination of fecal samples revealed that about 40 % of patients were asymptomatically colonized with toxigenic *C. difficile* at the start of transplant hospitalization [34]. *C. difficile* infection occurred in this subset of pre-colonized patients, suggesting that the high rates of infection are not well explained by nosocomial (hospital-acquired) transmission.

A subsequent study of this cohort [35] compared microbiome profiles of patients who developed clinical infection with those of asymptomatic carriers without clinical infection, using a time series modeling approach. Results from this study showed protective effects from *Clostridium scindens*, a non-pathogenic intestinal species within the bacterial family Lachnospiraceae (Clostridium cluster XIVa). In the same study, colonization of mice with *C. scindens* conferred protection against *C. difficile* [35]. It was further shown that the likely mechanism of protection occurs through production of secondary bile acids, which inhibit vegetative growth of *C. difficile* [36, 37]. Results from other microbiome studies have also provided evidence that Lachnospiraceae confers protective effects against *C. difficile* infection by promoting colonization resistance [38].

Bacteria from the Bacteroidetes phylum also appear to have durable protective effects against *C. difficile* infection; in patients with recurrent *C. difficile* infection who were cured using fecal microbiota transplantation (FMT), examination of the microbiota before and after FMT revealed that the most obvious microbial change was significant colonization with Bacteroidetes, where it had been previously completely lacking [39, 40]. Further evidence can indirectly be seen with fidaxomicin treatment, which was shown to be non-inferior to oral vancomycin for the treatment of *C. difficile* infection, but with fewer observed recurrences [41]. This is hypothesized to be related to fidaxomicin’s narrower spectrum of activity; a previous study suggested that this drug spares *Bacteroides* spp. during treatment [42].

Given the high rates of *C. difficile* infection in at-risk populations such as HSCT patients, FMT and fidaxomicin treatment have both been raised as possible therapeutic strategies to prevent this complication during cancer treatment. Therapeutic clinical trials for both are ongoing [43, 44].

## Other microbiota links to cancer-related infections

### Infections outside the gut

The microbiome may influence risk for cancer-related infections at sites other than the intestinal tract. One recent study examining the impact of the gut microbiome on lung complications in recipients of allogeneic HSCT showed that disruption of the microbiota and overgrowth and domination by Gammaproteobacteria was associated with an increased risk of subsequent pulmonary complications [45]. The reasons for this association are still unclear; these findings may be due to bacterial translocation to the lungs during early HSCT or increased inflammation signaled by an aberrant gut or lung microbiome.

### Anatomic disruptions that affect microbiota compositions

In cancer, mechanical defects in intestinal anatomy are not uncommonly encountered. These may be caused by locally infiltrating cancer itself, radiation damage, or surgical interventions performed as part of cancer treatment. The impact of these anatomic derangements on the composition of the microbiota is unknown, but could have relevance to the overall outcome for these patients.

In patients with ileostomy or colostomy, the gut microbial composition has been studied and noted to be much more predominantly aerobic [46]. In small bowel transplant patients, presence of a temporary ileostomy was associated with a more dramatic shift in microbiota than small bowel transplant itself [47]. Beneficial anaerobes such as *Bacteroides* and *Clostridia* were largely missing in patients with ileostomy, and instead the gut microbiotas of these patients were dominated by facultative anaerobes [47]. Presumably this is related to increased oxygen content in the bowel following ileostomy. In this study, metabolomic profiling further showed increased metabolites derived from the Krebs cycle. It is unclear what the implications of this compositional shift are; the authors noted cases of sepsis due to enteric pathogens in patients with ileostomy [47]. If it is true that a colonic shift away from obligate anaerobic bacteria imparts increased risk of domination by potential pathogens and subsequent systemic infection in these patients, a re-evaluation of the indications for ileostomy might be considered.

## Balancing of antibiotics in cancer

Over the course of cancer treatment, antibiotics are administered frequently. Given the increased susceptibility of cancer patients to infection, antibiotic treatments may entail prolonged courses or may involve agents with a broad spectrum of activity, given either as treatment or as prevention in a high-risk patient. The heavy use of antibiotics in cancer care is likely to make the microbiome particularly clinically relevant in these patients.

The gut microbiome works to prevent infection by contributing to colonization resistance against pathogens and by stimulating host immune responses to infection. Paradoxically, although antibiotics are given to combat infection, these treatments can serve to harm natural host defenses against infection by disrupting beneficial bacteria that previously supported these host defenses. Early microbiome studies of healthy volunteers have suggested that even short courses of antibiotics can have a substantial impact on the gut microbiome [48]. With careful stewardship, however, antibiotics are still an essential part of patient care in current medicine.

Realizing that antibiotics remain a necessary evil, it is useful to note that antibiotics vary greatly in terms of their spectrum of activity not only against pathogens, but also against non-pathogenic beneficial microbes. For example, in recipients of allo-HSCT, metronidazole administration was associated with an increase in the abundance of intestinal VRE, which in turn preceded systemic infection with VRE in the setting of neutropenia and mucosal barrier injury [12]. However, ciprofloxacin administration successfully prevented an increase in the number of pathogenic Gram-negative bacteria such as Enterobacteriaceae, without significant disruption of healthy anaerobes, such as Clostridia or Bacteroides, which contribute to colonization resistance and protection against increasing numbers of pathobionts [12, 49, 50].

In addition to the spectrum of activity, antibiotics may differ greatly in terms of impact on gut microbiota because of penetration and route of administration. For instance, vancomycin administered orally remains confined to the gut, with little to no systemic absorption, and it has been observed to have profound inhibitory impact on beneficial gut microbes, including Bacteroidetes and other anaerobic bacteria [51]. In contrast, vancomycin given intravenously penetrates poorly into the gut lumen [52] and, therefore, has far less impact on the intestinal microbiota than when administered orally. Indeed, both microbiome studies and previous clinical studies have found no association between administration of intravenous vancomycin and colonization or infection with VRE, despite concerns to the contrary [12, 53, 54].

Based on these observations, each antibiotic’s spectrum of activity and pharmacologic distribution in the body clearly are important determinants of its impact on the microbiome. Given that antibiotics can range greatly from having profound deleterious effects on the microbiome to having little to no impact, antibiotics should be more clearly and precisely characterized as to their effect on the microbiota and clinicians should incorporate this knowledge into their therapeutic considerations.

## Conclusions and future steps

These studies suggest that the microbiome is an essential mediator in various infections encountered in the cancer setting. A normally functioning microbiota establishes an intricate relationship with its host, creating stability and preventing infection by promoting colonization resistance; however, these microbial populations can be completely disrupted with cancer treatment, giving rise to susceptibility for infection by opportunistic pathobionts.

Microbiome studies of cancer patients will lead to a better understanding of the role of the microbiota in cancer-related infections and will provide insight into how therapeutic interventions might be designed to exploit the benefits of commensal and symbiotic bacteria. For example, further studies should be done to explore the use of ‘microbiota-sparing’ antibiotics, which can effectively prevent or treat infections that arise during cancer treatment but at the same time preserve beneficial microbes that enhance host defenses and promote colonization resistance against infection. In addition, repair of damaged microbial populations through interventions such as FMT or bacteriotherapy should also be further explored to improve defenses in cancer patients where treatment-related disruption of the microbiome may be unavoidable. These approaches have been proposed as interventions that could be performed safely and effectively [55, 56]. An enhanced understanding of the microbiome will allow us to improve our management of cancer-related infectious complications.

## Abbreviations

Allo-HSCT: allogeneic hematopoietic stem cell transplantation
FMT: fecal microbiota transplantation
VRE: vancomycin-resistant *Enterococcus*
